# Male-Biased gga-miR-2954 Regulates Myoblast Proliferation and Differentiation of Chicken Embryos by Targeting *YY1*

**DOI:** 10.3390/genes12091325

**Published:** 2021-08-27

**Authors:** Xiuxue Dong, Yu Cheng, Lingyun Qiao, Xin Wang, Cuiping Zeng, Yanping Feng

**Affiliations:** Key Laboratory of Agricultural Animal Genetics, Breeding and Reproduction of Ministry of Education, College of Animal Science and Technology, Huazhong Agricultural University, Wuhan 430070, China; dongxiuxue@163.com (X.D.); chengyu20210713@163.com (Y.C.); 18202737453@163.com (L.Q.); zhdxz1314@163.com (X.W.); zcp115@mail.hzau.edu.cn (C.Z.)

**Keywords:** gga-miR-2954, *YY1*, myoblast, proliferation, differentiation

## Abstract

Previous studies have shown that gga-miR-2954 was highly expressed in the gonads and other tissues of male chickens, including muscle tissue. *Yin Yang1 (YY1)*, which has functions in mammalian skeletal muscle development, was predicted to be a target gene of gga-miR-2954. The purpose of this study was to investigate whether gga-miR-2954 plays a role in skeletal muscle development by targeting *YY1*, and evaluate its function in the sexual dimorphism development of chicken muscle. Here, all the temporal and spatial expression profiles in chicken embryonic muscles showed that gga-miR-2954 is highly expressed in males and mainly localized in cytoplasm. Gga-miR-2954 exhibited upregulated expression of in vitro myoblast differentiation stages. Next, through the overexpression and loss-of-function experiments performed in chicken primary myoblasts, we found that gga-miR-2954 inhibited myoblast proliferation but promoted differentiation. During myogenesis, gga-miR-2954 could suppress the expression of *YY1*, which promoted myoblast proliferation and inhibited the process of myoblast cell differentiation into multinucleated myotubes. Overall, these findings reveal a novel role of gga-miR-2954 in skeletal muscle development through its function of the myoblast proliferation and differentiation by suppressing the expression of *YY1*. Moreover, gga-miR-2954 may contribute to the sex difference in chicken muscle development.

## 1. Introduction

In vertebrates, skeletal muscle formation is a complex process that involves a series of steps during embryogenesis, including the activation of satellite cells into muscle precursor cells (myoblasts), the withdrawal of myoblasts from the cell cycle, the differentiation of myoblasts into myotubes, and ultimately forming the mature muscle fibers. For the whole myogenic progression, the key to that regulatory process is the sequential activation of myogenic regulatory factors (MRFs: *MYOD (myogenic differentiation 1), MYF5 (myogenic factor 5), MYOG (myogenin), MRF4 (Myf6, Herculin)*) [1,2,3] and the cofactor of myocyte enhancer factor 2 (MEF2) which is necessary for the expression and activation of MRFs during myoblast differentiation [4]. Besides MRFs and MEF2, many other factors and signal pathways are involved in this regulation process, for example, the PAX and Six family, the signal pathways of Wnt, TGF-β and Notch, and non-coding RNA like lncRNAs (Long noncoding RNAs) and miRNAs (MicroRNAs) [5]. These regulatory factors interact with each other, and finally form a comprehensive network to regulate muscle development in an orderly manner.

MiRNAs are small non-coding RNAs of approximately 22 nucleotides that post-transcriptionally regulate gene expression. MicroRNAs are involved in many physiological processes by regulating downstream target genes, such as tissue development, cell proliferation and differentiation, cell apoptosis, energy metabolism and carcinogenesis. Frequent evidence indicates that miRNAs have an important role in the development of skeletal muscle. In Dicer knockout mice, skeletal muscle dysplasia and perinatal death occurred [6]. Recently, miRNA transcriptome analysis of human myoblasts showed that at least 60 miRNAs were differentially expressed during myogenic differentiation [7]. Goljanek-Whysall et al. [8] summarized several miRNAs that can be regulated by muscle transcription factors in the process of skeletal muscle formation in mammals. Among those miRNAs, miR-206, miR-1 and miR-133 have been widely studied and best characterized in skeletal muscle differentiation [9,10,11,12]. In birds, recent studies have shown that miR-223 [13] and miR-133a-3p [14] can accelerate chicken primary myoblast from the proliferative phase to the differentiation phase, whereas, miR-203 [15], miR-16-5p [16], miR-133a-5p/miR-29b-1-5p [17] and miR-130b-3p/301b-3p [18] inhibits myoblast differentiation. These results further revealed the wide participation of miRNAs in the regulation of the growth and development of skeletal muscles.

Gga-miR-2954, as an avian specific miRNA, is expressed at significantly higher levels in males than in females in brain, gonad, heart, liver, and muscle tissues of adult zebra finches, which suggest that it might play a role in sexually dimorphic development and function in avian species [19]. In our previous study, we demonstrated the male-biased expression of gga-miR-2954 in all detected tissues of E10.5 chicken embryos (unpublished). In addition, some studies reported that gga-miR-2954 may mediate some of the song-responsive neurogenic effects [20,21] as well as the brain perception, discrimination and memory related to song which have sexual dimorphisms [22]. Gga-miR-2954 was differentially expressed in the immune response in cecum of broilers infected with salmonella and treated with probiotic, which suggested that gga-miR-2954 might participate in the immune regulation of cecum in chicken although no target immune genes were found [23]. Therefore, gga-miR-2954 might be extensively involved in the sexual dimorphic development and maintenance of sex differences in multiple tissues.

*Yin Yang1 (YY1)* is expressed ubiquitously in various tissue types and modulates a variety of biologic processes, in particular, the development of muscle, nerve, immune cells/tissues and tumorigenesis in which it is a key regulator in cell proliferation, differentiation and apoptosis [24]. Intensive efforts in the past decade underscore the role of *YY1* and enhance the knowledge with respect to how *YY1* acts in skeletal muscle formation and muscle regeneration. The study of skeletal-muscle-specific *YY1* knockout (YY1mKO) mice showed that its inactivation might contribute to exercise intolerance and mitochondrial myopathies [25]. When myogenesis begins, Linc-YY1 interacts with *YY1*, then YY1/PRC2 (Polycomb repressive complex) needs to be released and replaced by the MyoD/PCAF/SRF complex, thus activating the expression of myofibrillar genes [26,27]. In addition, Linc-YY1 also regulates PRC2-independent function of *YY1* in skeletal myogenesis [28]. However, there lacks verified information on the role of *YY1* gene in chicken myoblast proliferation and differentiation, and whether its role in chicken skeletal muscle is regulated by miRNAs.

Sexual dimorphism in body weight and muscle mass between male and female chickens has attracted much attention, but the regulatory mechanism remains unknown. Given the results of previous studies that relate to sex-specific expression of gga-miR-2954 in chicken muscle and the role of its predicted target *YY1* in mammalian skeletal muscle development, we hypothesized that this miRNA could exert its role in chicken skeletal myogenesis by targeting *YY1*. In this study, we demonstrated the hypothesis by further charactering the expression of gga-miR-2954 in chicken embryonic muscle and investigating the regulatory function of miR-2954 and *YY1* on the proliferation and differentiation of chicken myoblasts. Our results provide novel evidence for further exploring the potential regulatory mechanisms of gga-miR-2954 in the formation of sex differences in chicken muscle development.

## 2. Materials and Methods

### 2.1. Animals and Tissue Collection

All the animal experiments in this study were performed in accordance with the Guidelines for the Care and Use of Laboratory Animals and approved by Huazhong Agricultural University (Wuhan, China). The fertilized eggs of Jianghan chickens (an indigenous breed in Hubei province, China) were provided by Hubei Kangjian Poultry Development Co., Ltd. and incubated at 37.8 °C and 60–70% relative humidity in an incubator. Chicken embryos at different stages were used for leg muscle collection for RNA extraction, DNA extraction, Fluorescence in situ hybridization (FISH) and primary myoblast culture. For RNA extraction, the leg muscle of each chicken embryo was harvested separately from the egg at embryonic Day 7 (E7), E10, E13, E15, E18.5 and birth (D1) and stored in RNA safer for further use. The male leg muscle of E13.5 chicken embryos were isolated and fixed in 4% paraformaldehyde for at least 48 h at 4 °C and then 4-mm paraffin sections prepared for FISH. DNA was extracted from the mesentery of embryos at E7 for sexing by PCR [29] and the gender of each embryo at E10-D1 was clearly distinguished by its gonad morphology.

### 2.2. Cell Isolation and Culture

The chicken embryos at embryonic Day 10.5 (E10.5) were used to prepare chicken primary myoblasts (CPMs) as described previously [12]. Firstly, the leg muscle was separated from chicken embryos and the skin and bones removed. These pooling muscles from 20 to 30 chicken embryos were then physically cut up with scissors. After that, tissue was digested with trypsin (Invitrogen, Carlsbad, CA, USA) at 37 °C for 20 min and intermittently shaken to release cells. The growth medium was used to neutralize and stop digestion. Next, the suspension was filtered with a 400m Cell Strainer (Sorfa, China) and centrifuged at 1000 rpm, 10 min to enrich cells. The CPMs were cultured in growth medium (DM) with a formula: Roswell Park Memorial Institute (RPMI)-1640 medium (Gibco, New York, NY, USA) supplemented with 20% fetal bovine serum (FBS) (Gibco) and 1% penicillin-streptomycin Solution (Invitrogen) with 5% CO_2_ atmosphere at 37 °C. Differentiation medium (DM) was used to induce differentiation of myoblasts by replacing 20% fetal bovine serum with 2% horse serum (HyClone, Logan, UT, USA).

The DF-1 cell line of chicken embryonic fibroblast (ATCC, Manassas, VA, USA) was cultured in DMEM (Gibco) supplemented with 10% fetal bovine serum (FBS, Gibco) and 1% penicillin-streptomycin Solution (Invitrogen) followed by incubation at 37 °C in 5% CO_2_ for the following experiments.

### 2.3. RNA Oligonucleotides, Plasmids Construction and Transfection

The RNA oligonucleotides including gga-miR-2954 mimics, inhibitors, and their corresponding negative controls (NC mimics and NC inhibitor) as well as YY1-siRNA (the sequences are shown in Table 1) were all synthesized by GenePharma (Shanghai, China). The full-length cDNA of *YY1* was synthesized and cloned into pcDNA3.1 plasmid (Invitrogen) to make the overexpression vector pcDNA3.1-YY1. The wide-type (WT) and mutant segments of *YY1* 3’UTR were amplified and cloned into the psiCHECK-2–reporter vector (Promega, Madison, WI, USA) to make psiCHECK^TM^2-YY1 3’UTR recombinant plasmids.

These reagents were transfected into cells with the help of Lipofectamine 2000 (Invitrogen) following the instruction of the manufacturer. The concentrations used for above reagents transfections were as follows: plasmid, 2 µg /mL (the concentration of plasmid is proportional to the amount of medium); mimics, a concentration of 20 nM; inhibitors and siRNA transfection with a concentration of 100 nM.

### 2.4. RNA Extraction, RT-PCR and Quantitative Real-Time PCR

The muscle tissues and the cells were treated with TRIzol reagent (Invitrogen) to extract total RNA following the standard protocol, and only RNAs with a 260/280 ratio of 1.8–2.1 were used for further experiments after being measured using the NanoDrop 2000 (Thermo Scientific). The PrimeScript RT reagent kit with gDNA Eraser (Takara, Otsu, Japan) was used to synthesize complementary DNA (cDNA) according to the manufacturer’s suggestion. Quantitative real time PCR was performed in the QuantStudio™ 6 Flex Real-Time PCR System (ABI, Waltham, USA) using iTaqTM Universal SYBR^®^ Green Supermix (Bio-Rad, Hercules, CA, USA). Relative expression levels of miR-2954 and tested genes, which were calculated using the 2^−ΔΔCt^ method, were normalized to the expression of 5S rRNA (for miRNAs) and *GAPDH* (for mRNAs), respectively. All primer sequences are shown in Table 1.

### 2.5. Western Blot

The total proteins were extracted from cells, which were dissolved with RIPA (Radio-Immunoprecipitation Assay) Lysis buffer (Beyotime, Shanghai, China) and 1% PMSF (Phenyl methane sulfonyl fluoride) (Beyotime). Protein samples with a loading buffer were electrophoresed on sodium dodecyl sulfate-polyacrylamide gel electrophoresis (SDS–PAGE), then transferred to a polyvinylidene difluoride (PVDF) membranes (Millipore, Boston, MA, USA). After blocking by 5% skimmed milk and subsequently incubating with primary antibody anti-desmin antibody (D33, ab8470, Abcam, LDN, USA) (1:1000), MyoD (SPM427, Novus, USA) (1:500), YY1 (ab43058, Abcam) (1:1000) and GAPDH (Beyotime) (1:2000) at 4 °C overnight, and the corresponding secondary antibody [horseradish peroxidase-conjugated goat anti-rabbit or goat anti-mouse secondary antibodies (Beyotime) (1:2000)] at room temperature for 1 h. The blots were exposed to ECL reagent (Thermo Scientific, Waltham, MA, USA) and detected by ImageQuant LAS4000 (GE Healthcare, Chicago, IL, USA).

### 2.6. Fluorescence In Situ Hybridisation Assay

The anti-gga-miR-2954 probe and a scrambled probe were synthesized by Guangzhou Exon Biotechnology Inc (Guangzhou, China). The D-R type in situ hybridization kit (D-2810B) (Exon Biotechnology Inc, Guangzhou, China) was used to treat the 4-mm thick paraffin section from E13.5 chicken leg muscle followed the manufacturer’s protocol. In detail, the paraffin section was treated with Xylene and absolute ethanol to perform the dewaxing process, the samples need to be fixed in 4% paraformaldehyde after treating with Liquid A and Liquid B at room temperature for 20 min and 15 min, respectively. Next, the slides were pre-hybridized with 100μL miRNA Hybridization Buffer at 55 °C for 1 h. The probes were incubated with miRNA Hybridization Buffer (1:100) at 85 °C for 3 min, then added to the slides for incubation at 42 °C for 24 h. After washing twice with washing buffer, the slides were incubated with Anti-Digoxin Rhodamine-Conjugate and Blocking Buffer I (1:100) in the dark at 37 °C for 1 h and DAPI for 20 min at room temperature. Finally, the images for detection of the expression of digoxigenin labelled probe were captured using an Olympus microscope (Tokyo, Japan).

### 2.7. Dual-Luciferase Reporter Assay

In 24-well plate, chicken DF-1 cells were co-transfected with 100ng of luciferase reporter vector, YYl-3’UTR-psiCHECK^TM^2 wild-type plasmid (YY1-WT) or YYl-3’UTR-psiCHECK^TM^2 mutant plasmid (YY1-Mut) and either 50 nmol/L gga-miR-2954 mimics or NC mimics per well using Lipofectamine 2000 transfection reagent (Invitrogen). After transfection for 48 h, the activities of firefly and Renilla luciferase were measured using a dual luciferase reporter assay system (Promega) at PerkinElmer Enspire (PerkinElmer, Waltham, MA, USA). The value of firefly luciferase signal was normalized to that of the renilla luciferase signal, and the normalized activity was compared among different groups.

### 2.8. CCK-8 (Cell Counting Kit 8) Assays

CCK-8 assay was used to detect cell proliferation. About 1.0 × 10^3^ myoblasts per well were seeded in 96-well plates, transfected and cultured for 0 h, 24 h, 36 h and 48 h respectively. Next, cells in each well were incubated with 10 μL CCK-8 solution (Biosharp, Suzhou, China) for 2 h and measured the absorbance at 450 nm using PerkinElmer EnSpire.

### 2.9. EdU (5-Ethynyl-2’-deoxyuridine) Assays

The cell proliferation was performed using the EdU assay kit (Ribobio, Guangzhou, China) according to the manufacturer’s instructions. Briefly, the transfected myoblasts were exposed to the medium supplemented with 10 μM EdU for over 24 h. Then, cells were fixed with 4% paraformaldehyde and permeabilized with 0.5% Triton X-100. Subsequently, cells were incubated in 1× Apollo reaction solution for 30 min and stained with Hoechst 33342 for 30 min, and the views were captured by Nikon TE2000 microscope (Nikon, Tokyo, Japan).

### 2.10. Immunofluorescence Analysis

Differentiated myoblasts were cultivated in 10-mm dishes and used to perform immunostaining assay. The transfected cells were fixed in 4% formaldehyde for 20 min, followed the permeabilization with 0.5% Triton X-100 for 10 min and blocking with 1% bovine serum albumin (BSA) for 1 h. Besides, the next steps were incubation with primary antibody (anti-MyHC) (bs-9862R, Bioss, Beijing, China) (1:100) overnight at 4 °C, fluorescent secondary antibodies (FITC-conjugated anti-rabbit IgG) (1:200) at 37 °C for 1h and DAPI (Beyotime) for 5 min at room temperature. Ultimately, images were viewed using a confocal laser scanning microscopy (Zeiss LSM800, Göttingen, Germany).

### 2.11. Statistical Analysis

All statistical results showed in columns were represented as mean ± SEM from at least three independent experiments. Student’s t-test was used for *P*-value calculations.

## 3. Results

### 3.1. Expression Profiles and Localization of gga-miR-2954 in Chicken Embryonic Skeletal Muscle

**Figure 1:**
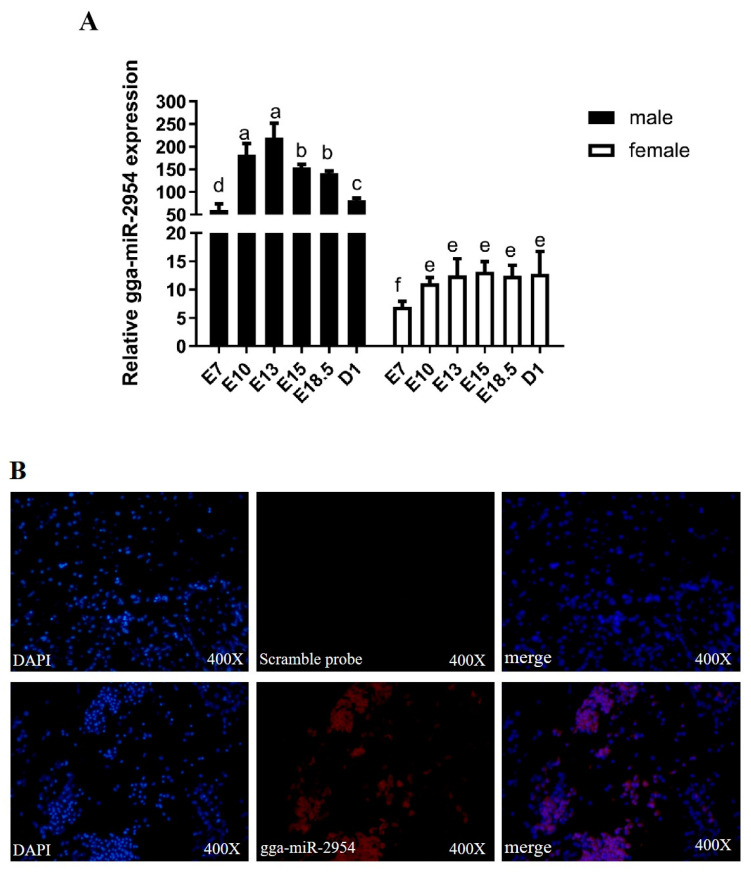
The expression of gga-miR-2954 during the development of chicken embryonic skeletal muscle. (**A**) Gga-miR-2954 expression in chicken embryonic leg muscle between sexes from E7-D1 stages. Bars with different superscripts are significantly different (*p* < 0.05). (**B**) Fluorescence in situ hybridization result of gga-miR-2954 in male leg muscle at E13.5, red staining indicates gga-miR-2954-positive hybridization signals.

The expression of gga-miR-2954 during embryonic muscle development at six stages from E7 to D1 was measured by Q-PCR. Results showed that it was highly expressed in males but very low in females. Specifically, in males, the expression of gga-miR-2954 was steadily increased from E7 and remained at a significantly high level from E10 to E18.5 and then dramatically decreased, while its expression was kept at a low level in female at all detected stages (E10-D1) (Figure 1A). In order to further explore the localization of gga-miR-2954 in chicken muscle tissue, its expression in E13.5 male chicken leg muscle was detected by fluorescence in situ hybridization and it was found that positive hybrid signals of gga-miR-2954 were abundant in the cytoplasm, with little in the nucleus (Figure 1B). These results suggest that gga-miR-2954 might play a role in skeletal muscle development.

### 3.2. Expression Profiles of gga-miR-2954 during Chicken Myogenesis

**Figure 2:**
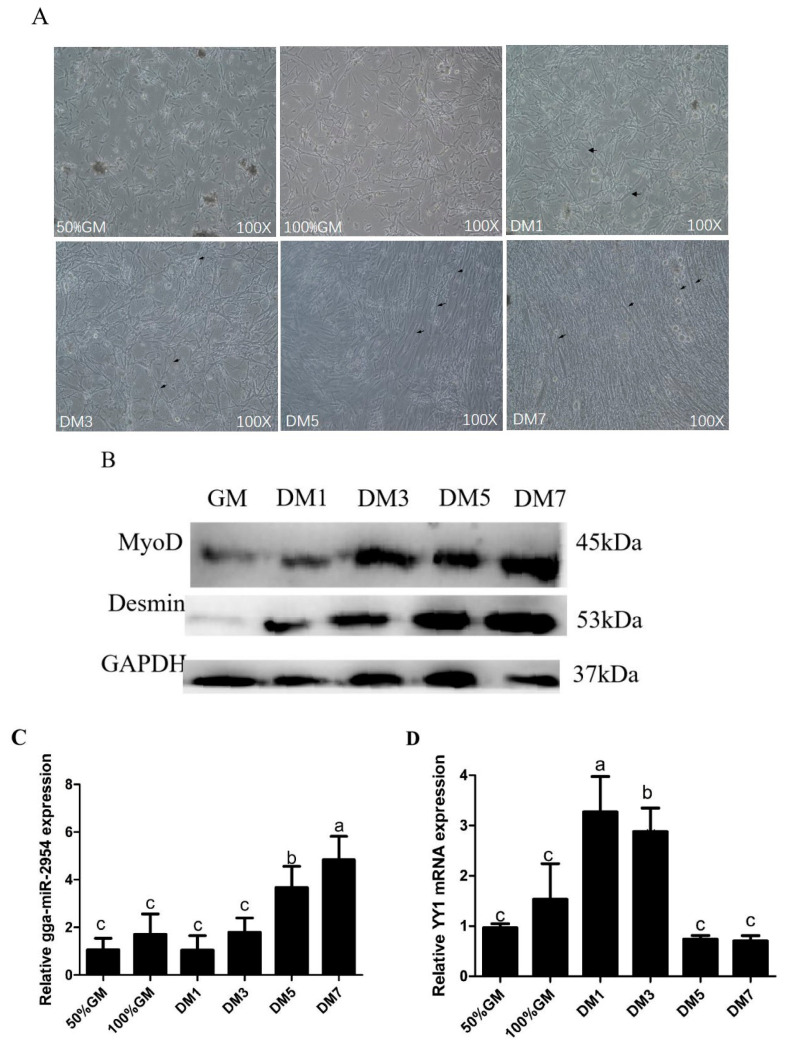
The expression of gga-miR-2954 and *YY1* during chicken primary myoblast differentiation. (**A**) The morphological map of chicken primary myoblasts during proliferation (50% and 100% growth density, 50% GM and 100% GM) and differentiation from the 1st to 7th day (DM1 to DM7). Black arrow indicates the myotube. (**B**) The protein expression of MyoD and Desmin. (**C**) Q-PCR analysis of gga-miR-2954 expression in myogenesis in vitro. (**D**) The mRNA expression of *YY1*. All measurements shown were the means ± SEM from at least three independent experiments, and the different lower-case letter indicates significant difference, *p* value < 0.05.

We cultured chicken primary myoblasts in vitro and induced the differentiation process for 1, 3, 5, and 7 days (DM1, DM3, DM5, DM7) from proliferation (50% and 100% growth density, 50% GM and 100% GM) (Figure 2A). In the proliferative stages, the number of cells was obviously increased, and no myotube formation was found. In the differentiated stages, there was only a small scattered myotube formation seen in the first day of differentiation (DM1), a relatively thick myotube formation in DM3, interwoven and similar to shape of muscle fibers myotubes were seen after DM5. According to Western blot analysis, the expression of MyoD and Desmin, two myoblast differentiation markers, increased as differentiation progressed (Figure 2B). We further assessed the expression profiles of gga-miR-2954 and *YY1* during chicken primary myogenesis in vitro, and results showed that the expression levels of gga-miR-2954 significantly increased after the differentiation stage of DM3 (Figure 2C), while *YY1* was significantly decreased (Figure 2D).

### 3.3. Gga-miR-2954 Inhibits Myoblast Proliferation

**Figure 3:**
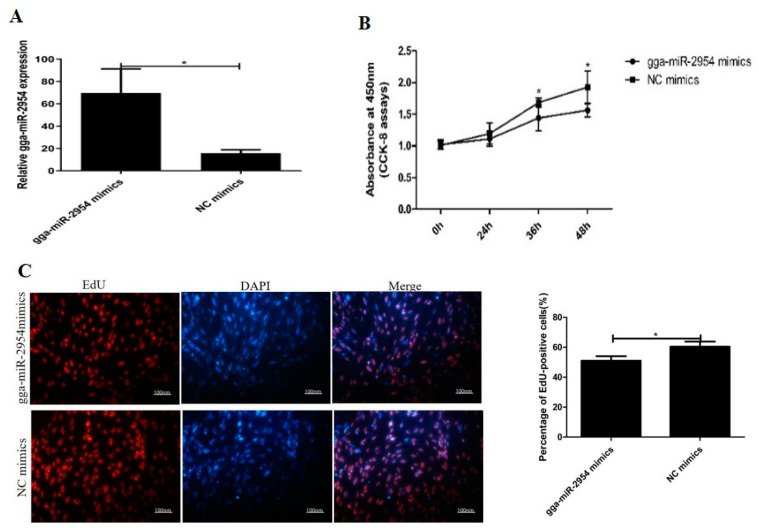

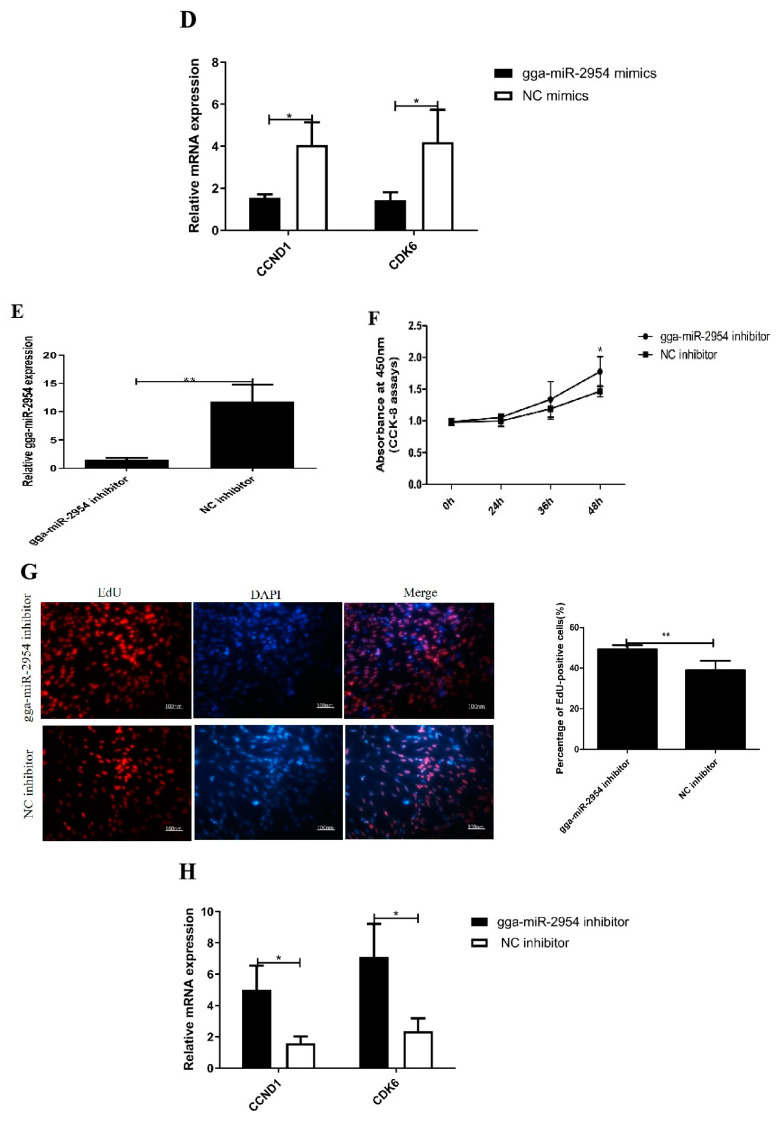
Gga-miR-2954 suppresses myoblast proliferation. (**A**) The overexpression efficiency of gga-miR-2954 after transfection of mimics. (**B**) Cell proliferation rate after gga-miR-2954 overexpression detected by CCK-8. (**C**) EdU cell viability test after gga-miR-2954. overexpression. Scale bar = 100 μm. (**D**) The mRNA expression of *CCND1* and *CDK6* after transfection of gga-miR-2954 and NC mimics. (**E**) The expression of gga-miR-2954 after its inhibitor transfection. (**F**) Cell proliferation rate after gga-miR-2954 and NC inhibitor transfection detected by CCK-8. (**G**) EdU cell viability test after transfection of gga-miR-2954 inhibitor and NC inhibitor. (**H**) The mRNA expression of *CCND1* and *CDK6* after transfection of gga-miR-2954 inhibitor and NC inhibitor. All values were presented as the mean ± SEM from at least three independent experiments (* *p* < 0.05; ** *p* < 0.01).

To observe the effects of gga-miR-2954 on myoblast proliferation, we transfected myoblasts with gga-miR-2954 mimics and its negative control (NC), and gga-miR-2954 mimics markedly increased the expression level of gga-miR-2954 (Figure 3A). By monitoring the proliferation status of cells using CCK-8 assay, the detection results showed that gga-miR-2954 overexpression reduced the cell number significantly after transfection for 36 h and 48 h (Figure 3B). In addition, EdU assay indicated that the ratio of EdU-staining cells was significantly decreased in gga-miR-2954 mimics transfected cells compared with that in control cells (Figure 3C). Moreover, the mRNA expression of cell cycle regulatory factors such as *CCND1* and *CDK6* was notably downregulated when over-expressing gga-miR-2954 (Figure 3D). Similarly, we transfected CPMs with gga-miR-2954 inhibitor or NC-inhibitor to study the effect of gga-miR-2954 loss-of-function on proliferating myoblasts (Figure 3E). Compared with NC-treated cells, the total number of cells in the gga-miR-2954 inhibitor group was increased significantly at 48 h (Figure 3F), and the number of EdU-staining cells were also increased (Figure 3G). Furthermore, the qPCR analysis revealed that the inhibition of gga-miR-2954 promoted the expression of *CCND1* and *CDK6* (Figure 3H). Together, these data demonstrated that gga-miR-2954 could repress chicken primary myoblast proliferation.

### 3.4. Gga-miR-2954 Promotes Myoblasts Differentiation

**Figure 4:**
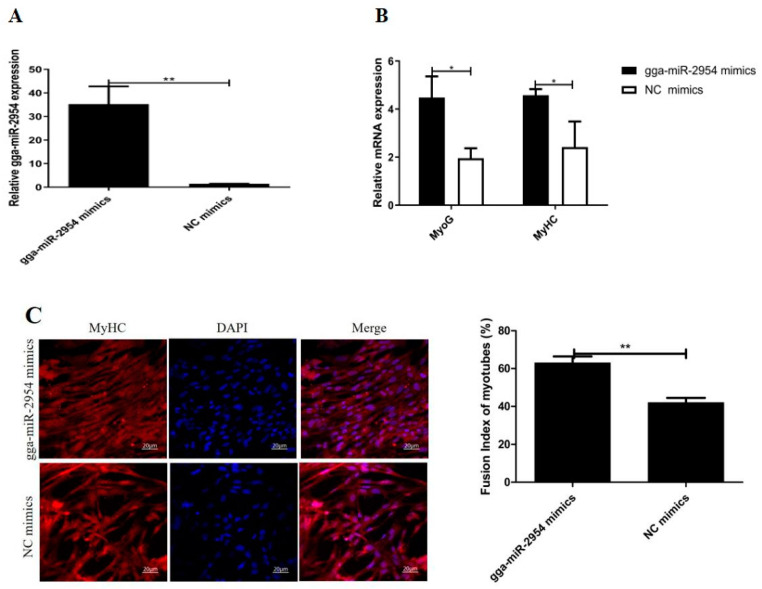

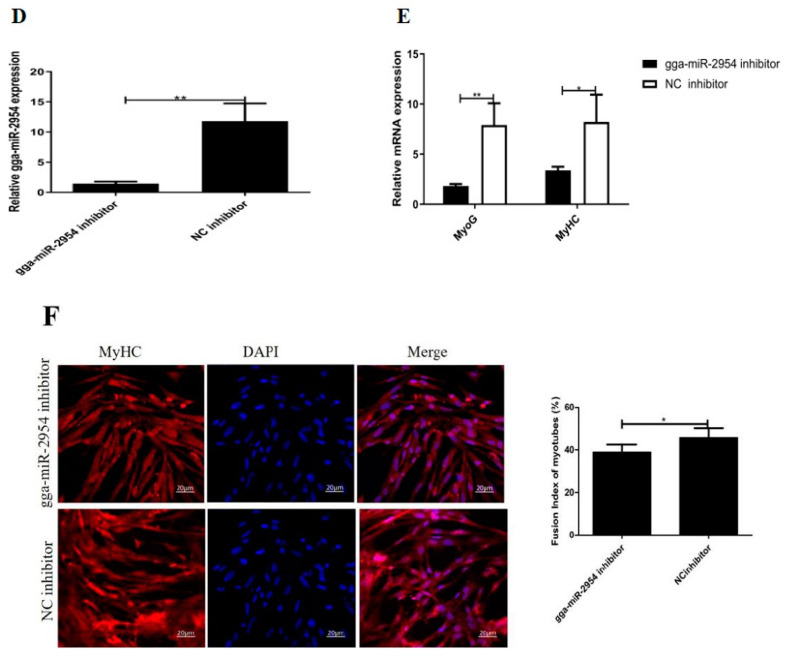
Gga-miR-2954 promotes myogenic differentiation. (**A**) The expressive efficiency of gga-miR-2954 mimics. (**B**) The mRNA expression of *MyoG* and *MyHC* after transfection with gga-miR-2954 mimics or NC mimics. (**C**) The results of MyHC immunofluorescent staining after transfection with gga-miR-2954 and NC mimics. Bar = 20 μm. (**D**) The expression of gga-miR-2954 after its inhibitor transfection. (**E**) The mRNA expression of *MyoG* and *MyHC* after gga-miR-2954 inhibitor and NC transfection. (**F**) The results of MyHC immunofluorescent staining after gga-miR-2954 inhibitor and NC transfection. All results were presented as the mean ± SEM from at least three independent experiments (* *p* < 0.05; ** *p* < 0.01).

Myoblasts withdraw from cell cycle progression and subsequently step into the differentiation process. Hence, we further analyzed the roles of gga-miR-2954 in primary myoblast differentiation. Firstly, we transfected gga-miR-2954 or NC mimics into CPMs and found that gga-miR-2954 expression was markedly up-regulated in differentiated cells (Figure 4A). By qPCR analysis, we discovered that the overexpression of gga-miR-2954 significantly promoted the mRNA expression of the myogenic marker genes such as *MyoG* and *MyHC* (Figure 4B). Next, the immunofluorescence results of MyHC showed an upward trend in the amount and size of myotubes in the gga-miR-2954 mimics group compared with that in NC mimics group (Figure 4C). Besides, we detected the effect of gga-miR-2954 loss-of-function on myoblast differentiation (Figure 4D). The results showed that gga-miR-2954 inhibitor suppressed the relative expression of *MyoG* and *MyHC* (Figure 4E). Moreover, the myotube status of differentiated cells treated with gga-miR-2954 inhibitor was dramatically repressed relative to that treated with NC inhibitor (Figure 4F). Collectively, these findings suggest that gga-miR-2954 exerts a positive regulatory effect during the primary myoblast differentiation.

### 3.5. Gga-miR-2954 Targets YY1 during Myoblast Proliferation and Differentiation

**Figure 5:**
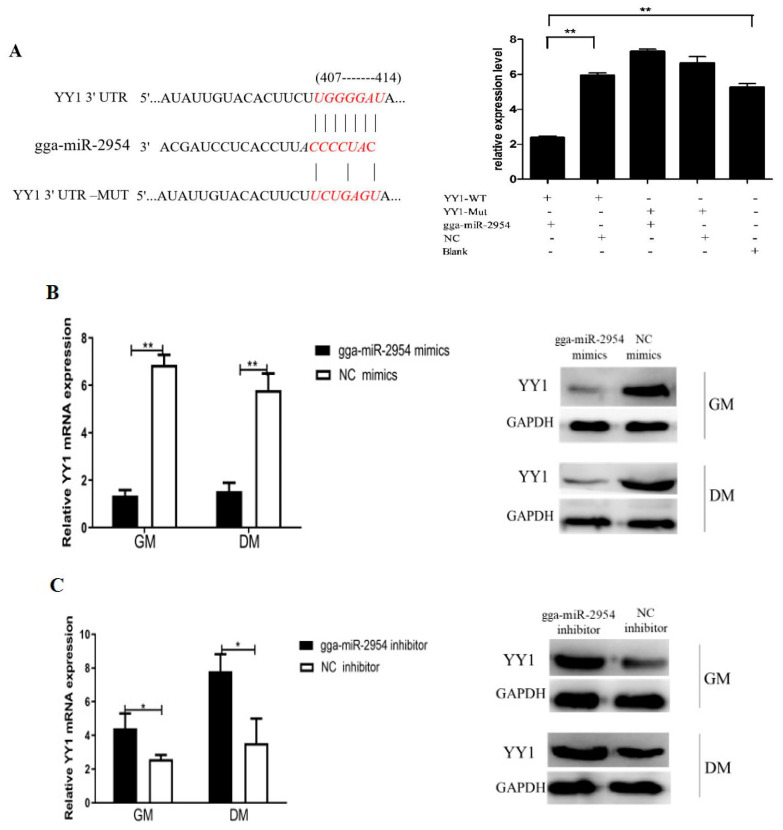
Gga-miR-2954 targets *YY1* during myoblast myogenesis. (**A**) Luciferease reporter constructs based on the potential binding site of gga-miR-2954 with the 3’ UTR of *YY1* and the detection of the relative luciferase activities in DF-1 cells. WT, wild-type. Mut, mutant-type. (**B**) The mRNA and protein expression of YY1 after transfection with gga-miR-2954 mimics. (**C**) The mRNA and protein expression of YY1 after transfection with gga-miR-2954 inhibitor. All measurements shown are the means ± SEM from at least three independent experiments (* *p* < 0.05; ** *p* < 0.01).

*YY1* has been predicted as the target gene of gga-miR-2954 by the TargetScan 7.2 database. Here their targeted relationship was further verified by performing a dual-luciferase reporter assay in chicken DF-1 cells. The two recombinant reporter vectors, YY1-WT and YY1-mutant dual-luciferase reporter were co-transfected with gga-miR-2954 mimics or NC into DF-1 cells respectively, and the results showed that overexpression of gga-miR-2954 effectively suppresses the luciferase activity of YY1-WT compared to the YY1-mutant reporter (Figure 5A). Additionally, for myoblasts transfected with gga-miR-2954 mimics in proliferation and differentiation phases, the mRNA and protein levels of YY1 were markedly downregulated when compared with that in the NC mimics group (Figure 5B), while inhibition of gga-miR-2954 upregulated the expression of YY1 both at transcription and translation (Figure 5C). These results indicated that gga-miR-2954 negatively regulates the expression of *YY1* during myogenesis.

### 3.6. Gga-miR-2954 Regulates Myoblast Proliferation by Targeting YY1

**Figure 6:**
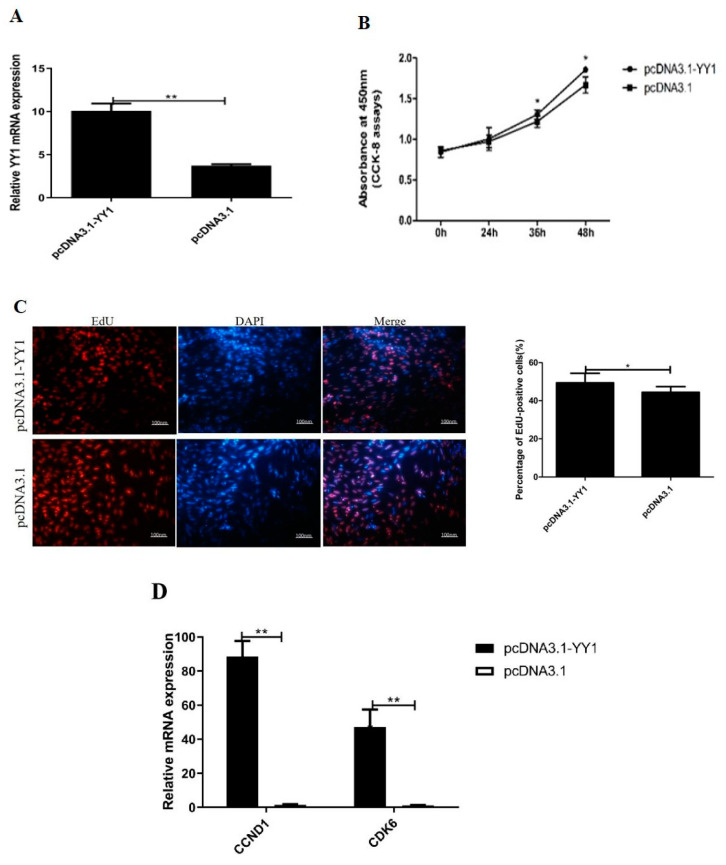

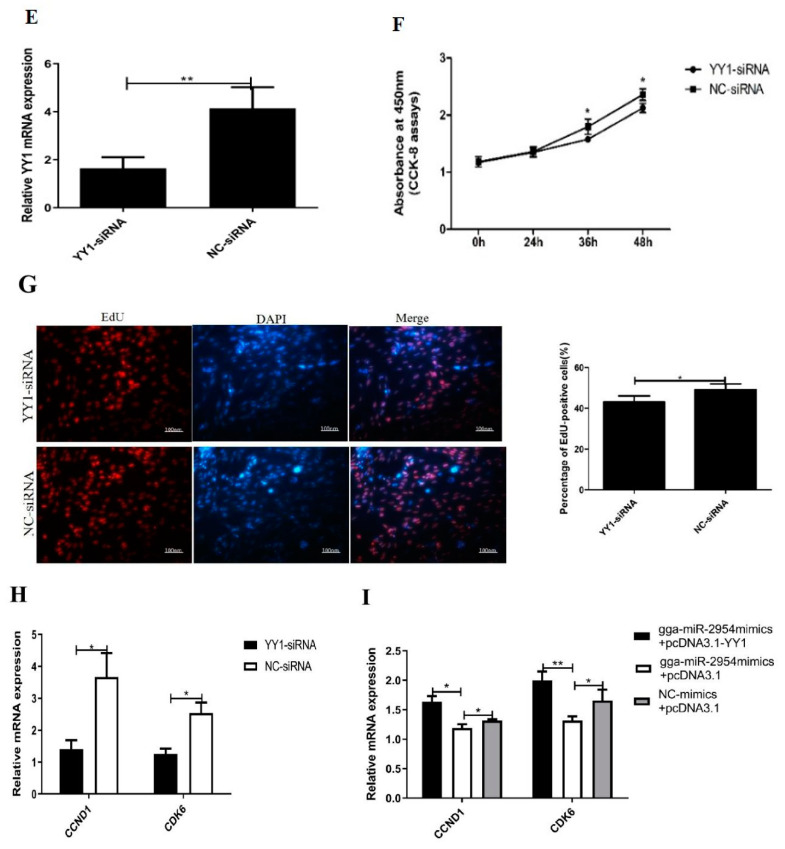
Gga-miR-2954 inhibits myogenic proliferation through targeting *YY1*. (**A**) The mRNA expression of *YY1* after pcDNA3.1-YY1 transfection. (**B**)The results of cell proliferation test of CCK-8 after *YY1* overexpression. (**C**) The results of EdU cell viability test for *YY1* overexpression. Scale bar = 100 μm. (**D**) The mRNA expression of *CCND1* and *CDK6* after *YY1* overexpression. (**E**)The mRNA expression of *YY1* afterYY1-siRNA transfection. (**F**)The results of cell proliferation test of CCK-8 afterYY1-siRNA transfection. (**G**) The results of EdU cell viability test for *YY1* inhibition. (**H**) The mRNA expression of *CCND1* and *CDK6* after YY1-siRNA transfection. (**I**)The mRNA expression of *CCND1* and *CDK6* after gga-miR-2954 and pcDNA3.1-YY1 transfection. Data represent the mean ± SEM from at least three independent experiments (* *p* < 0.05; ** *p* < 0.01).

To further study the potential role of *YY1* in myoblast proliferation, we used pcDNA3.1-YY1 plasmid transfection to elevate the expression of *YY1* (Figure 6A) and detected its effect on cell proliferation and gene expression compared with pcDNA3.1 treatment. The result of the CCK-8 assay demonstrated that the total number of cells increased significantly with pcDNA3.1-YY1 treatment after 36 h and 48 h (Figure 6B), and the EdU staining results indicated that the pcDNA3.1-YY1 treated group had a higher proportion of proliferating cells than the pcDNA3.1 group (Figure 6C). Moreover, overexpression of *YY1* significantly increased the relative expression of cell cycle-related genes *CCND1* and *CDK6* (Figure 6D). In contrast, *YY1* loss-of-function was obtained by YY1-siRNA (Figure 6E), the total number of cells decreased in the YY1-siRNA group according to the CCK8 assay (Figure 6F), and a lower proliferation rate of EdU-staining cells in YY1-siRNA group than that in NC-siRNA group (Figure 6G). Moreover, the interference of *YY1* markedly hampered the mRNA expression of *CCND1* and *CDK6* (Figure 6H). In addition, we co-transfected gga-miR-2954 mimics and pcDNA3.1-YY1 in proliferating myoblasts, and found that the level of *CCND1* and *CDK6* were downregulated in gga-miR-2954 overexpression myoblasts but the suppressive effect could be overcome by *YY1* overexpression (Figure 6I). Together, these results indicated that gga-miR-2954 can regulate myoblast proliferation through suppressing *YY1*.

### 3.7. Gga-miR-2954 Regulates Myoblast Differentiation by Targeting YY1

**Figure 7:**
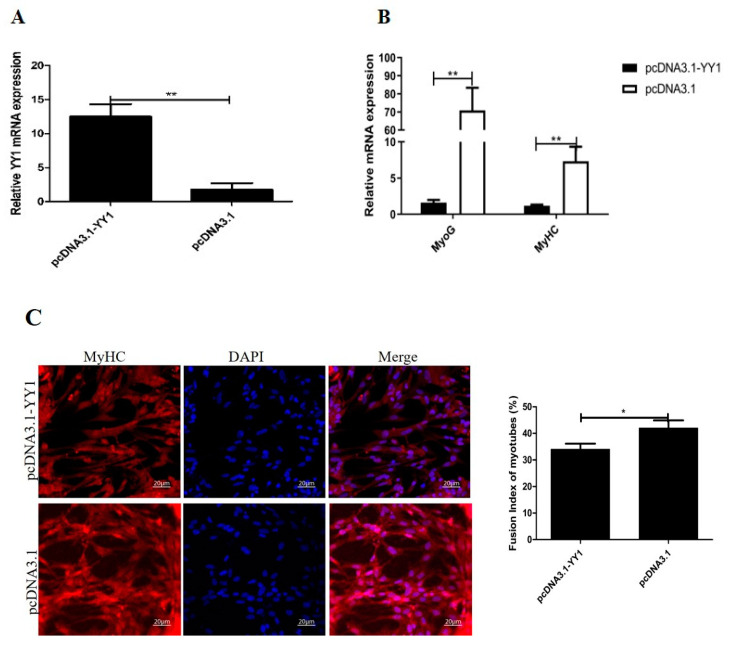

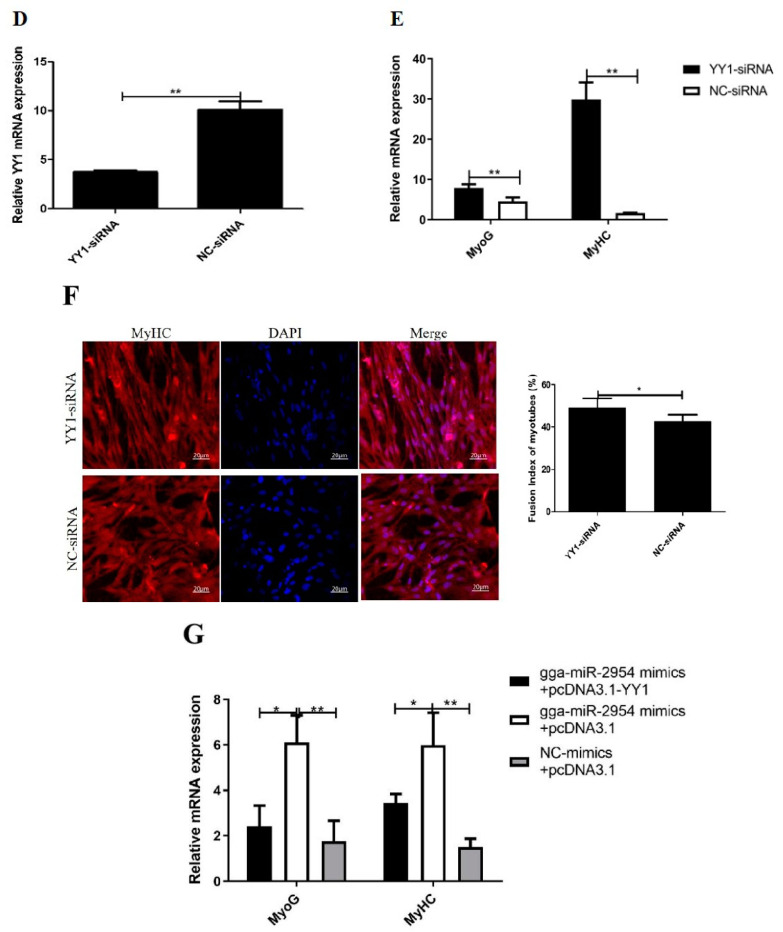
Gga-miR-2954 promotes myogenic differentiation by targeting *YY1*. (**A**) The expression efficiency of pcDNA3.1-YY1 in differentiated myoblasts. (**B**)The mRNA expression of *MyoG* and *MyHC* after *YY1* overexpression. (**C**) The results of MyHC immunofluorescence staining after *YY1* overexpression. Scale bar = 20 μm. (**D**)The expression efficiency of YY1-siRNA in differentiated myoblasts. (**E**)The mRNA expression of *MyoG* and *MyHC* after *YY1* inhibition. (**F**) The results of MyHC immunofluorescence staining after *YY1* inhibition. (**G**) The effect of co-transfection of gga-miR-2954 and *YY1* on the expression of myoblast differentiation related genes, *MyoG* and *MyHC*. Values represent the mean ± SEM from at least three independent experiments (* *p* < 0.05; ** *p* < 0.01).

After being transfected with pcDNA3.1-YY1 plasmid in differentiated cells, *YY1* mRNA expression of myoblasts was markedly upregulated (Figure 7A). By qPCR analysis, both the mRNA expression of the two myogenic marker genes, *MyoG* and *MyHC*, showed significantly decreased profiles in the pcDNA3.1-YY1 transfected cells compared to control cells (Figure 7B). Meanwhile, the myotube formation status detected by immunostaining assay demonstrated that overexpression of *YY1* inhibited the differentiation of myotubes (Figure 7C). Afterwards, we also conducted *YY1* loss-of-function experiments in myoblasts by YY1-siRNA, and the mRNA level of *YY1* was significantly inhibited as shown in Figure 7D. It was also found that inhibition of *YY1* significantly promoted the expression of *MyoG* and *MyHC* genes (Figure 7E) as well as myotube formation (Figure 7F) when compared to control treatment. In addition, we co-transfected gga-miR-2954 mimics and pcDNA3.1-YY1 in differentiated myoblasts, and found that YY1 was able to inhibit the upregulated expression of myogenic markers *MyoG* and *MyHC*, which were induced by the overexpression of gga-miR-2954 (Figure 7G). Thus, we testify that miR-2954 regulates chicken primary myoblast differentiation by inhibiting YY1.

## 4. Discussion

Our present study firstly investigated the expression pattern of gga-miR-2954 during embryonic skeletal muscle development and found it plays a vital role in regulating chicken myoblast proliferation and differentiation. In both birds and mammals, skeletal muscle development begins at the embryonic stage and includes two steps of primary development and secondary development. In chicken, primary fibers are formed within 6 days of hatching, and secondary fibers are formed along with the formation of separate myotubes, which begin to differentiate at 12–16 days from the hatching period [30]. The number of muscle fibers is basically fixed in the last embryonic stage, and it will not increase after hatching or birth. The muscle growth after birth is mainly due to the thickening and hypertrophy of muscle fibers [31]. It is well known that sex differences on growth rate and muscle development occur in most animals. An earlier report has demonstrated the differences of muscle characteristics between sexes in chicken embryos, with larger myofibers in female muscles, and more, but smaller, myofibers seen in male muscles [32]. Preliminary studies have shown that sex differences in muscle development may be related to sex hormones, but this point of view wasn’t fully support by the research of androgen or anti-androgen injection which only affects single sex individuals [33,34]. It is necessary to clarify the mechanism of gender differences on chicken muscle development by gene regulation. In this study, we confirmed the male-biased feature of gga-miR-2954 in the developmental progress of embryonic skeletal muscle. Specifically, in males, gga-miR-2954 showed high expression from 10 to 18.5 d of the hatching phrase, which consistent with the process of muscle fiber transformation from primary to secondary muscle fibers in chicken embryos. It is suggested that gga-miR-2954 might be involved in the regulation of skeletal muscle development in male embryos.

Based on the temporal expression profile of gga-miR-2954, FISH on leg muscle tissue section of male chicken embryo at E13.5 was performed to examine its spatial expression. It was found that gga-miR-2954-positive hybrid signals were abundant in cytoplasm and a little in the nucleus, which is consistent with the biosynthesis of miRNAs. In animals, the primary miRNAs (pri-miRNAs) are successively processed by Drosha into precursor miRNAs (pre-miRNAs) within the nucleus, but pre-miRNAs must be transported into the cytoplasm and finally converted into mature miRNAs by Dicer. The mature miRNAs are important regulators and involved in many physiological processes through the regulation of downstream target genes in the cytoplasm. In this study, we verified that gga-miR-2954 can bind to the 3’UTR of *YY1* by the dual-luciferase reporter assay in DF-1 cells, and gga-miR-2954 can directly target *YY1* and suppress the expression of *YY1* in the myoblast proliferation and differentiation stages. Intensive efforts in the past decade have shown that *YY1* acts as a repressive factor to inhibit myogenesis in mammals [24] and the high expression of gga-miR-2954 in male chicken muscle, leads us to speculate that gga-miR-2954 might be involved in the biological process of muscle development by suppressing the expression of *YY1*.

The proliferation and differentiation of embryonic myoblasts largely determines the number of muscle fibers, thus affecting muscle production. We conduct a series of experiments, and the results show that gga-miR-2954 can inhibit myoblast proliferation and promote the expression of myogenic genes *MyoG* and *MyHC*, which differentiate into myofibers [35], thus, we speculate that in the process of muscle fiber formation in the chicken embryo, gga-miR-2954 might accelerate the differentiation process of myoblasts by allowing myoblasts to exit from the cell cycle and quickly enter into the differentiation process. Gga-miR-2954 is involved in the development of chicken skeletal muscle, which provides new evidence for our supposition that miR-2954 might participate in the development and maintenance of multiple organs. In addition, the temporal and spatial expression profiles of gga-miR-2954 in chicken embryo muscle showed that gga-miR-2954 was preferentially expressed in male, which was also consistent with the previous reports in zebra finch. All of these are beneficial to support the hypothesis that miR-2954 might be involved in the sexual dimorphic development and maintenance of multiple tissues.

In skeletal muscle development, *YY1* is considered to play a negative role in myogenesis because *YY1* is stimulated by NF-κB and inhibits myofibrillar promoters by recruiting Ezh2 as well as the histone deacetylase protein HDAC-1 [36]. Numerous *YY1* binding sites are found on other myofibrillar promoters/enhancers, raising the possibilities that *YY1* regulates the myofibrillar genes [37]. Such a notion is also supported by *YY1* knockout mice. A large number of muscle-specific genes were derepressed after depletion of *YY1* in embryonic fibroblast cells [38]. In our present study, we found that the expression of *YY1* decreased significantly after the 5th day of myoblast differentiation in vitro, which confirmed the previous report that *YY1* mainly plays a role in myogenesis by directly repressing the expression of late-stage differentiation genes, including α-skeletal actin, muscle creatine kinase (*MCK*), and myosin heavy chain IIb (*MyHCII*b) [36]. Furthermore, a series of in vitro experiments also demonstrated that *YY1* can significantly promote the proliferation and inhibit differentiation of myoblasts, which is consistent with the above reports.

Some studies have reported the regulatory circuitry functions of miRNAs with *YY1* during skeletal myogenesis. In mice, miR-29, miR-1 and miR-34c can promote myogenic differentiation by targeting *YY1*, while *YY1* in turn downregulates both miR-29 and miR-1 [37,39,40]. Given a study reported that the prevalence of *YY1* binding sites in the genome, i.e., about 70% of vertebrate genes and 24% of viral genes contained *YY1* binding elements [41], many researchers speculated that other miRNAs could be regulated by *YY1* and forming numerous functional regulatory circuits. Our results confirmed that gga-miR-2954 regulates myoblast proliferation and differentiation by targeting *YY1* through co-transfection assay. Further evidence is required to find whether the feedback loop exists between gga-miR-2954 and *YY1*.

## 5. Conclusions

In summary, our study revealed a novel mechanism of gga-miR-2954 in regulating myogenesis by inhibiting myoblast proliferation and promoting myoblast differentiation, and it exerts biological effects through targeting *YY1*. It is noteworthy that the significantly male-biased expression of gga-miR-2954 might result in the higher degree of myoblast differentiation and more muscle fibers in male chicken embryos. However, the complex regulatory mechanism of gga-miR-2954 mediating skeletal muscle development in chicken remains to be further explored.

## Figures and Tables

**Table 1:** Related primers used in Q-PCR.

Primers/Oligonucleotides	Sequences (5′–3′)
gga-miR-2954	GGTAGGCATCCCCATTCCACTC
	AACTGGTGTCGTGGAGTCGGC
*YY1*	AGGGACAACTCTGCTATGA
	AACGCTTTCCACATCCT
*CDK6*	CCAGACCCGCACAACCTATT
	TCTTGGCTGGATTGAACGCT
*CCND1*	ATAGTCGCCACTTGGATGCT
	AACCGGCTTTTCTTGAGGGG
*MYOG*	CGGAGGCTGAAGAAGGTGAA
	CGGTCCTCTGCCTGGTCAT
*MyHC*	CTCCTCACGCTTTGGTAA
	TGATAGTCGTATGGGTTGGT
*MYOD*	GCTACTACACGGAATCACCAAAT
	CTGGGCTCCACTGTCACTCA
*GAPDH*	GAGGGTAGTGAAGGCTGCTG
	CACAACACGGTTGCTGTATC
5S rRNA	CCATACCACCCTGGAAACGC
	TACTAACCGAGCCCGACCCT
gga-miR-2954 probe	TGCTAGGAGTGGAATGGGGATG
Scramble Probe	GTGTAACACGTCTATACGCCCA
gga-miR-2954 mimics	CAUCCCCAUUCCACUCCUAGCA CUAGGAGUGGAAUGGGGAUGUU
NC mimics	UUCUCCGAACGUGUCACGUTT ACGUGACACGUUCGGAGAATT
gga-miR-2954 inhibitor	UGCUAGGAGUGGAAUGGGGAUG
NC Inhibitor	CAGUACUUUUGUGUAGUACAA
YY1-siRNA	AGAAGCAGGTGCAGATCAA

## Data Availability

Not applicable.
